# The Preparation of the Mouth for a Denture

**Published:** 1898-09

**Authors:** Robert H. Nones

**Affiliations:** Philadelphia.


ARTICLE IV.

### THE PREPARATION OF THE MOUTH FOR A DENTURE.

ROBERT H. NONES, D. D. S., PHILADELPHIA.

Read at a meeting of the Academy of Stomatology, January 25th, 1898.

In offering, this evening, this paper, it is not with the idea of presenting any originality whatever, but rather of calling attention to a part of that branch of dentistry which is seemingly much neglected—namely prosthesis.

We must observe carefully the condition of the parts, whether normal or abnormal; discover, if possible, the cause of the abnormal conditions, and remove or treat them. We must give particular attention to the extraction or retention of teeth or roots, and at the same time plan the future denture, mapping out what to make and how to make it.

In the examination of an edentulous mouth notice the general shape, whether it be regular or irregular, the vault high or low, the ridge prominent or flat. Note carefully any hard or bony protuberances in the vault of the palate, as well as an irregularly resorbed ridge of either the upper or low jaw.

If the soft or flabby conditions of the tissues exist, proper relief or pressure must be given. Location of the muscles must be carefully observed, as it is of vital importance to the proper outlining of the plate, attention to which gives comfort to the patient. All these conditions have considerable bearing on the planning of the denture.

Another equally important condition is that of a fissure of the vault, starting in the soft palate and extending at times quite far into the hard palate. This is often so narrow in appearance as to deceive one as to its depth, except on close examination. These fissures run usually through the centre of the mouth, but may be found double, one on either side of the hard central prominence,

Frequently unsuccessful dentures would have been successful had proper cognizance been taken of this condition. The knowledge of its existence will enable us to avoid a space between the denture and the mouth, and to exclude the air which would otherwise enter.

Inflamed or irritated mouths, referring for the present to edentulous mouths, may result from various causes, both local and systemic.

Dyspepsia plays an important part, and its treatment would naturally be relieved by systemic treatment.

The use of ardent spirituous liquors and of tobacco may also cause conditions of irritability.

The most frequent cause of these inflamed or irritated conditions, and one which should be looked for, is a previously worn denture. How frequently will a patient, when we are about to make an examination, quickly remove from the mouth a denture, and with an expression of shame carefully close it from view in a napkin or in a handkerchief. The cause of the embarrassment generally is the filth.

Another cause, less frequent, but not at all infrequent, is the adaptation of foreign substances, such as wax, cotton, and even chewing-gum to ill-fitting dentures. In this connection I recall an incident showing apparently how an otherwise cleanly person may be unconscious of the actual condition of the mouth.

A gentleman, about sixty years of age, his general appearance denoting refinement, neatness, and cleanliness, sought my services to have a partial lower metal clasp piece repaired. One of the natural teeth had become quite loose, and he wished an artificial one attached to the plate in its stead. On examination I noticed it was rather a difficult piece to remove, and told him to remove it, as I wished to save him the inconvenience which is often occasioned by the removal by the dentist of such a piece. I shall never forget his look of mingled surprise and helplessness when he told me it had never been removed from the time it was first inserted, some five years previously.

I removed the piece with some difficulty, making no effort to hurry it past his nostrils during the withdrawal from the mouth, which caused him, as I had anticipated, to withdraw his head. It was foul enough and covered with calculus and debris. The discomfiture of the patient was marked, and not at all feigned. He informed me that he always believed himself to be cleanly, bathing at least once daily and changing his under-garments as precisely. His mortification was intense.

While we should naturally expect human beings to be cleaner in the mouth than any other part, it is our duty always to instruct them in regard to the proper care and removal ot dentures.

Frequently we find the mouth irritated from roughness on the plate. In some cases there are prominences on so-called temporary plates which were formerly seated in tooth-sockets; in other cases sharp, rough, angularly outlined, and deep vacuum-chambers, or perhaps too highly outlined margins which impinge on the muscles and mucous membrane, cutting them and creating irritable wounds, especially in lower dentures. How can we expect success to follow the making of a denture on such faulty foundations.

The treatment in such cases is simple. First comes the removal of the cause. If the denture be a metal one, carefully boil it in a dilute solution of sulphuric acid; on removal, allow it to cool, then wash with soap and water, and place in a solution of soda, thus neutralizing any acid beneath the teeth, after which any roughness should be removed and sharp or rough edges of vacuum-chamber dressed down and smoothed. The plate should then be brushed with a stiff lathe-brush and pumice, then polished and carefully washed.

The treatment of a vulcanite denture is the same, with the exception that it is allowed to stand in a cool solution of hydrogen dioxid in place of warm sulphuric acid.

Small pin-point prominences may be looked for, caused by the vulcanite forcing into the pores or small bubble-like places on the surface of the plaster, these are frequently a source of great irritation and annoyance to a patient, and they can be largely avoided by using a tin model instead of a plaster one. When they exist in a plate they should be removed.

The denture after proper treatment, providing it fits well and is not likely to cause further irritation, may be worn till the mouth is in condition for the impression, otherwise its use must be discontinued.

The medicinal treatment of the effects produced by the roughness consists in the application of antiseptic mouth-washes, such as "listerine," "borine," etc. The most effectual and quickest treatment I have found is the painting of the inflamed parts with tincture of iodin, together with the frequent use of a 3 per cent. solution of hydrogen dioxid by the patient as a mouth-wash.

During the examination of the mouth not infrequently will be found prominent points of the alveolar process, due to uneven resorption after the extraction of the teeth. These should be removed.

The attachment of the frenum of the lip to the alveolar process frequently being very low, it may interfere with the upper margin of the denture, or we may find what is known as a double lip or a false lip, which is caused by an ill-fitting plate or one extending too high. Sometimes this extends below the natural lip. These with the excessively flabby conditions may receive surgical treatment.

What is of less frequent occurrence is the prominence of the alveolar process. I recall one which I was fortunate enough to see both before and after operation for its reduction. This was performed by Dr. M. H. Cryer. Any one on looking at the patient with her lips closed, which was a difficult act for her to do, would have concluded that her dentist had made a grave mistake in the fullness of her denture. Dr. Cryer removed about a quarter of an inch of the labial and buccal process, thus placing the mouth in a fit condition for an artificial denture.

Should the mouth not be an edentulous one, we may look for other causes of irritation.

One, and an important one, is salivary calculus, an irritant which should generally be removed. I have been very successful with the use of iodin in connection with the ordinary scaling method, which I follow up with and have the patient use freely the hydrogen dioxid.

In mouths containing some natural teeth we will frequently find partial dentures creating irritation of the gums about the necks of the teeth. A sinking in of the plate and a bulging out of the gum around and between the teeth and plate is caused most frequently by the plate not fitting accurately to the natural teeth.

The extraction or retention of teeth or roots depends on the advantages to be gained. When they cannot be made healthy they are to be extracted. To make a broad assertion, they are not to be extracted unless positively detrimental to the health of the patient and to the success of the denture. We must remember that an artificial tooth is not generally as valuabie as the natural organ. No tooth is to be extracted merely to facilitate the taking of an impression or the making of a denture. We should adopt ourselves to proper circumstances rather than adapt the circumstances to ourselves.

If all the four upper incisors are in place, they should be retained, other conditions permitting; as the cuspids can be artistically matched to them.

The cuspids should be kept whenever it is possible; even the roots should be preserved and crowned, as they not only retain that facial expression which it is almost impossible to regain with an artificial denture, but they also guide and stay the bite.

They should be extracted, however, when they cannot be brought to a healthy condition, or when so abnormally situated as to interfere with proper adjustment of a denture, as, for instance, when interfering with the bite or protruding to such a degree as to cause disfigurement, or if dropping inward, especially if occluding inside of the lower teeth; or if there be excessive recession of the gum, or a tilting from or toward the median line, thus making it impossible to properly adjust artificial teeth between the natural teeth; or if out of line from or toward the centre, not allowing sufficient space for the proper size or number of teeth; or if elongated, giving a marked canine expression to the face.

Bicuspids, except when on both sides of the mouth end useful as clasp attachments, should be extracted.

Molars should be retained whenever possible, as they afford a means of support by clasping in mouth, which are difficult to fit with vacuum plates. The roots should be preserved and crowned for clasp attachment.

In the lower jaw none of the teeth should be extracted when it is possible to retain them, with possibly the exception of a single central or lateral or laterals in pairs.

The other teeth affords a means of support for a denture till the patient may become accustomed to its management.

In extraction all sharp prominences of the alveolar process should be removed, thus permitting a smooth, even resorption.— *International.*

